# Effects of long-term indoor air purification intervention on cardiovascular health in elderly: a parallel, double-blinded randomized controlled trial in Hong Kong

**DOI:** 10.1016/j.envres.2024.118284

**Published:** 2024-04-15

**Authors:** Xi Xia, Ka Hung Chan, Timothy Kwok, ShaoWei Wu, Chung Ling Man, Kin-Fai Ho

**Affiliations:** a Department of Occupational and Environmental Health, School of Public Health, Xi’an Jiaotong University Health Science Center, Xi’an, Shaanxi, China; b Key Laboratory of Environment and Genes Related to Diseases, Ministry of Education, China; c School of Public Health, Shaanxi University of Chinese Medicine, China; d The Jockey Club School of Public Health and Primary Care, The Chinese University of Hong Kong, Hong Kong; e Clinical Trial Service Unit and Epidemiological Studies Unit, Nuffield Department of Population Health, University of Oxford, UK; f Oxford British Heart Foundation Centre of Research Excellence, University of Oxford, UK; g Department of Medicine and Therapeutics, The Chinese University of Hong Kong, Hong Kong, China; h The Jockey Club Centre for Osteoporosis Care and Control, The Chinese University of Hong Kong, Hong Kong, China

**Keywords:** Long-term, Indoor air purifier, Elderly, Cardiovascular health

## Abstract

Ambient fine particulate matter (PM_2.5_) is a leading environmental risk factor globally, and over half of the associated disease burden are caused by cardiovascular disease. Numerous randomized controlled trials (RCT) have investigated the short-term cardiovascular benefits of indoor air purifiers (IAPs), but major knowledge gaps remain on their longer-term benefits. In this 1-year, randomized, double-blinded, parallel controlled trial of 47 elderly (n_true-purification_ = 24; n_sham-purification_ = 23) aged ≥70 years, true-purification reduced household PM_2.5_ levels by 28% and maintained lower exposure throughout the year compared to the sham-purification group. After 12 months of intervention, a significant reduction of diastolic blood pressure was found in the true-purification versus sham-purification group (-4.62 [95% CI: -7.28, -1.96] mmHg) compared to baseline measurement prior to the intervention, whereas systolic blood pressure showed directionally consistent but statistically non-significant effect (-2.49 [95% CI: -9.25, 4.28] mmHg). Qualitatively similar patterns of associations were observed for pulse pressure (-2.30 [95% CI: -6.57, 1.96] mmHg) and carotid intima-media thickness (-10.0% [95% CI: -24.8%, 4.7%]), but these were not statistically significant. Overall, we found suggestive evidence of cardiovascular benefits of long-term IAPs use, particularly on diastolic blood pressure. Evidence on other longer-term cardiovascular traits is less clear. Further trials with larger sample sizes and long-term follow-up are needed across diverse populations to evaluate the cardiovascular benefits of IAPs.

## Highlights

• The cardiovascular impact of long-term indoor air purifiers (IAPs) is unknown.
• We conducted a 1-year randomized controlled trial on IAPs in ∼50 elderly.
• Long-term IAP use reduced indoor PM_2.5_ throughout the year and by ∼28% overall.
• IAP use was associated with significant reduction of diastolic blood pressure.

## Graphical abstract

**Figure:**
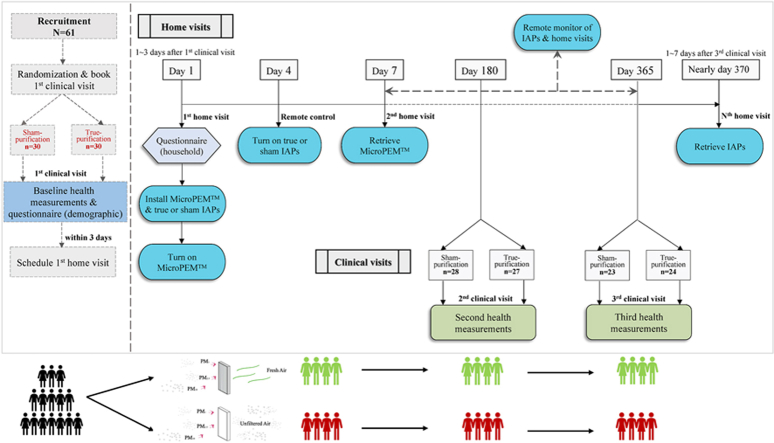

## 1 Introduction

Affecting over 90% of the world’s population (World Health Organization, 2021), ambient fine particulate matter (PM_2.5_) is a leading environmental risk factor, estimated to account for 4.1 million premature deaths and 118 million disability-adjusted life years (DALYs) globally in 2019 (GBD 2019 Risk Factors Collaborators, 2020). Ample epidemiological evidence have shown the linkage of PM_2.5_ to cardiovascular disease (CVD), which contributed to over half of the disease burden attributed to PM_2.5_ (GBD 2019 Risk Factors Collaborators, 2020).

Despite the increasing public concern about air quality, the progress to strengthen existing air pollution control policies remain slow in most populations (Rajagopalan et al., 2020). Moreover, as most people spend 70–90% of their time indoors (Chan et al., 2021; Klepeis et al., 2001), there has been an emerging recognition of the importance of indoor air quality (Sarah and Duffield, 2023), and rapidly growing interest in indoor air purifiers (IAP) to reduce indoor PM_2.5_ levels and mitigate health risks (Health Effects Institute, 2020; Rajagopalan et al., 2020). As such, a growing body of evidence from randomised controlled trials (RCTs) on the cardiovascular effects of IAP have emerged (Lin et al., 2011; Shao et al., 2017; Weichenthal et al., 2013; Xia et al., 2023). Recent systematic reviews and meta-analyses of these trials largely consistently reported IAP interventions resulted in significant reduction in indoor PM_2.5_ levels, systolic blood pressure (SBP), and pulse pressure (PP) (Liu et al., 2022; Xia et al., 2021). This is coherent with an emerging literature of controlled exposure and observational studies showing temporal association of PM_2.5_ exposure and changes in BP (Cosselman et al., 2012; Lin et al., 2022; Soppa et al., 2017). However, the reviews also identified substantial heterogeneity between studies, and all but one trial assessed only short intervention duration (mostly <14 days), leaving important uncertainties on the cardiovascular effects of long-term IAP use.

The only relevant long-term RCT involved a 1-year crossover intervention in 200 homemakers aged 28–61 years (mean: 43 years) (Chuang et al., 2017). The use of low-efficiency filters installed in air conditioners was associated with significant reduction of diastolic blood pressure (DBP, mean change [95% confidence interval (CI)]: -3.20 [-4.50, -1.90] mmHg) and SBP (-7.7 [-10.00, -5.40] mmHg). However, the employed intervention deviates from the typical use of high-efficiency particulate absorbing (HEPA) filters in IAPs, and the relevance of IAPs among elderly, who appeared more vulnerable to the harm of PM_2.5_ (Gouveia and Fletcher, 2000) and spend longer time indoors (Chan et al., 2021; Klepeis et al., 2001), remains unknown. Furthermore, to our knowledge, no previous trials have investigated the impact of IAPs on longer-term cardiovascular markers such as flow-mediated dilation (FMD) and carotid intima-media thickness (CIMT), which progress slowly and does not reverse easily. These indicators may shed light on whether reduced PM_2.5_ exposure via IAP use could slow down the progression of FMD or CIMT. We report herein a long-term RCT conducted to address the above knowledge gaps.

## 2 Materials and Methods

### 2.1 Study design and participants

This long-term, randomized, double-blind, parallel intervention trial was conducted from November 2017 to December 2020 in Hong Kong, a metropolis with considerable local and transboundary PM_2.5_ pollution (Luo et al., 2018). The sample size required (n=44 in total) was calculated based on the primary endpoint (i.e., SBP), using non-inferiority tests with 5% alpha, 80% power, and the effect sizes were obtained from a previous 1-year RCT (Chuang et al., 2017). Sixty-one elderly were recruited from the outpatient clinic in a major teaching hospital, the Prince of Wales Hospital (PWH). The inclusion criteria was an age of ≥70 years. The exclusion criteria included: 1) current smoker; 2) the presence of any smokers at home; 3) current use of IAP; 4) inability to give informed consent, and; 5) had lived in Hong Kong for <5 years. One participant quit before the randomization process, leaving 60 elderly from 56 households (56 single subjects and four couples) in the study. The study protocol was reviewed and approved by the Joint Chinese University of Hong Kong-New Territories East Cluster Clinical Research Ethics Committee. Written informed consent was obtained from all participants prior to participation.

**Figure 1:**
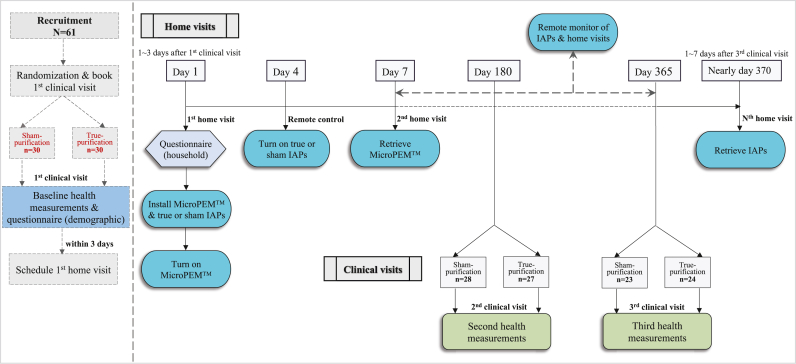
Flow chart of study procedures.

After recruitment, participants were randomly assigned into two groups, using computer-generated random numbers, to receive the true or sham HEPA IAP (Figure 1). Baseline health measurements, including BP, PP, FMD, and CIMT, were performed for each participant before any intervention. The first home visit was scheduled within three days after the baseline health measurements, during which a MicroPEM^TM^ sensor (Research Triangle Institute International, USA) and a true or sham IAP were installed, and a household environment questionnaire was administered by trained fieldworkers blinded to the intervention status. For the intervention group (i.e., true-purification group), a portable IAP (LIFAair LA352, Lifa Air Ltd, Finland) containing a HEPA filter (newly installed for each household) was used in the main activity room for 12 months. The clean air delivery rate (CADR) of this IAP is 332 m^3^/h for air particulates, which is suitable for rooms ranging from 23 m^2^ to 39 m^2^. The IAPs for the true-purification group were set to the automatic mode, enabling the devices’ fan speed to adapt to the real-time monitor of indoor PM_2.5_ (i.e., operate at a high speed when indoor PM_2.5_ levels were elevated, and vice versa). For the sham-purification group, the same model of IAP, with the same automatic fan setting but with the internal HEPA filter removed, was operated for 12 months for blinding purpose. At certain time periods, the automatic fan setting may result in minor differences the actual fan speed in the IAPs across the true- and sham-purification groups, as only the HEPA-IAPs could change from high to low speed after reducing the indoor PM_2.5_ to certain levels, but this is done in order to evaluate the most realistic usage of the IAPs.

During the 1-year intervention period, all participants were asked to keep the IAPs on all the time and keep the windows closed whenever possible, except when cooking. Both the true and sham IAPs were remotely monitored and controlled via the on-the-shelf LIFA APP, which allowed detection of abnormal operation (e.g., unexpected shut down, tempering of the filter or fan speed) that would prompt us to contact the subjects and, if necessary, attend the residence for maintenance. In addition to giving clear rules and information about not tempering with the IAP, we have stuck a tailored paper seal along the IAP filter replacement bracket to prevent and detect any tempering. No sign of abnormality was detected. The IAPs were retrieved within 7 days after the third clinical visit at the end of the 1-year intervention period, and a second household environment questionnaire was administered.

### 2.2 Outdoor and indoor exposure assessment

Outdoor monthly concentrations of PM_2.5_, PM_10_, nitrogen dioxide (NO_2_), ozone (O_3_), and sulphur dioxide (SO_2_) during each participant's intervention period were collected from their nearest fixed-sited monitoring stations (Norbäck et al., 2019), provided by the Environmental Protection Department. A total of 701 data points from 10 stations for 60 participants were collected.

Indoor PM_2.5_ concentrations in each household were continuously recorded by the built-in sensor in each IAP at an 1-hour resolution throughout the intervention period. To evaluate the particle removal efficiency of the air purifier, we co-located the IAP with a MicroPEM^TM^, an internationally validated sensor that can record real-time PM_2.5_ concentrations and collect gold-standard gravimetric samples (Cho et al., 2016; Du et al., 2019). Placing a MicroPEM^TM^ 1.5 m away from the IAP and 1.5 m above the floor, we obtained indoor PM_2.5_ data for three consecutive days before (i.e., unfiltered condition) and after (i.e., filtered condition) the IAP is turned on remotely on day-4.

On day-7, the MicroPEM^TM^ was retrieved and the internal PM_2.5_ filter was sent to the laboratory of the Chinese University of Hong Kong to be measured gravimetrically for manual recalculations. The PM_2.5_ concentrations recorded by the MicroPEM^TM^ sensor were calculated based on the net mass of the PM_2.5_ samples (i.e., ΔM, post-sample filter minus pre-sample filter), the total sample time (T), and the average flow rate (FR) by the equation $\frac{\Delta M}{\left(T\right)\left(FR\right)}$. All MicroPEM^TM^ filters were manually weighed, and the PM_2.5_ mass concentration was calculated and compared against the sensor readings of air purifiers for calibration. The calibration factor was derived from the fraction of gravimetric mass over the integrated mass estimates derived from the time-resolved data of MicroPEM^TM^, and all time-resolved readings were then adjusted by multiplying the calibration factor.

### 2.3 Health outcome measurements

Three clinical visits to the PWH were scheduled for each participant, with the first visit scheduled within three days before the installation of IAP, the second visit six months after the installation, and the third visit 12 months after the installation. For each clinical visit, height, weight, SBP, DBP, PP, and FMD were measured, while the CIMT measurements were only performed at the first and third visits. A questionnaire related to demographic information (e.g., age, gender, medical history etc.) was also administrated. All health outcome measurements were scheduled between approximately 09:00 and 12:00 before the participants had consumed any medicine, and all measurements were performed by trained personnel who were blinded to participants’ intervention status.

#### 2.3.1 Blood pressure and pulse pressure

After a rest of at least five minutes in a quiet room, BP measurements were taken from participants’ left upper arm using a digital autonomic BP monitor (Vital Signs Monitor, Heal Force Bio-Meditech Holdings Group, Shanghai, China). Three BP measurements, each separated by at least 2 min, were performed for each participant during each visit, and the average of the second and third readings was recorded. PP was estimated as the difference between DBP and SBP.

#### 2.3.2 Flow-mediated dilation and carotid intima-media thickness

FMD and CIMT measurements were performed using a high-resolution ultrasound scanner (SonoSite microMaxx, SonoSite Inc. Bothell, WA, USA) with a linear array 10 MHz transducer. After a supine rest for at least 5 min in a dimmed and quiet room, a pneumatic cuff was placed around the transducer on the upper left arm, and the ultrasound machine would scan and record the baseline diameter. The cuff was then inflated for 5 min (reaching at least 250 mmHg). The changes of artery diameter were continuously tracked until 60 s after the deflation of the pneumatic cuff, during which the maximum arterial diameter was recorded. All images were stored in an external hard disk for subsequent analysis.

FMD was calculated using established formulae: (maximum arterial diameter − baseline diameter)/baseline diameter (Thijssen et al., 2011). In each clinical visit, two FMD assessments were performed (10 min apart) for each participant, and the average of the two measurements was used for analysis. For CIMT, the machine would scan the far wall of the distal 10 mm of the common carotid artery (1 cm proximal to the bulb) for both the left and right sides. The final CIMT value was the mean value of both left and right CIMTs. A validated automatic edge-detection computer software package (M’Ath; Intelligence in Medical Technology, Paris, France) was used to digitize the images offline and detect the edges for obtaining the CIMT values.

### 2.4 Statistical analysis

Independent-sample Student’s t-tests and Fisher's exact tests were used to compare participants’ continuous and categorical baseline characteristics across the true- and sham-purification groups to check for balance. The differences between true- and sham-purification groups in the changes from baseline values to 6 months and 12 months were tested by one-way analysis of covariance (ANCOVA), with baseline value as covariate (i.e., SBP, DBP, PP, FMD, CIMT). The effects of the IAP intervention on the repeatedly measured health outcomes (i.e., SBP, DBP, PP, FMD) were estimated using linear mixed-effect (LME) models (Schultz et al., 2016; Wittkopp et al., 2016) with i) intervention status, age, gender, body mass index (BMI), baseline measured health outcome value, history of cardiovascular disease, and medicine use included as fixed effect parameters (Goldberg et al., 2015; Kioumourtzoglou et al., 2016; MiYang et al., 2014; Peacock et al., 2011) ii) participant ID as random effects to account for auto-correlation, and iii) a natural cubic spline function for the starting date of intervention with three degrees of freedom to account for the seasonality and long-term trend. The dependent variables in the models were the health outcomes measured in the 2^nd^ and 3^rd^ health assessments. The changes of health outcomes (i.e., SBP, DBP, PP, FMD) for an interquartile range (IQR) change in indoor PM_2.5_ were also estimated using LME models. There was no significant difference in outdoor air pollutants exposure (PM_2.5_, PM_10_, NO_2_, O_3_, SO_2_), so these were not included in the models. For CIMT, the treatment effects were analysed using analysis of covariance (Twisk et al., 2018), with log-transformed CIMT as the dependent variable and intervention status and the aforementioned participants characteristics as independent variables. All the data analyses were performed in the R software version 3.5.3.

## 3 Results

### 3.1 Participant characteristics

Of the 60 elderly randomised, five and eight quit before the second and third health assessments, respectively (Figure 1). Among the drop-outs, two were excluded because of the presence of smokers at home, two were dissatisfied with the noise of the IAPs, one reported no reason, six moved to nursing homes, and two died during the 1-year intervention. Finally, 47 subjects (n_true-purification_ = 24; n_sham-purification_ = 23) completed all procedures.

Comparing the true- and sham-purification groups, we found no statistically significant difference in the baseline non-clinical and clinical characteristics of participants who have completed at least two health measurements post-randomization (Table 1). A large proportion of the subjects had chronic diseases (e.g., hypertension, diabetes) and therefore took medications. Except for mean FMD, the baseline clinical characteristics appeared slightly lower in the sham-purification group, but the differences were not statistically significant.

**Table 1:** Basic characteristics of participants who have completed at least two health measurements post-randomization.

Characteristic	Sham-purification (n=28)	True-purification (n=27)	P value ^*a*^
***Non-clinical characteristic***			
Age, Mean ± SD	80.3 ± 5.5	81.8 ± 5.4	0.235
Female, n (%)	10 (35.7)	14 (51.9)	0.282
BMI, Mean ± SD	24.2 ± 3.7	23.9 ± 4.1	0.378
Living environment			
House area, Mean ± SD, m^2^	41.6 ± 16.4	42.1 ± 13.1	0.870
Keep pet, n (%)	1 (3.6)	2 (7.4)	0.611
Keep plant, n (%)	9 (32.1)	8 (29.6)	1.000
Household incense burning, n (%)	11 (39.3)	8 (29.6)	0.573
Cooking at home, n (%)			0.884
≥ 3 times per day	5 (17.9)	3 (11.1)	
1–2 times per day	21 (75.0)	22 (81.5)	
Rarely or never	2 (7.1)	2 (7.4)	
Cooking fuel use, n (%)			0.758
Natural gas	20 (71.4)	21 (77.8)	
Induction Cooker	8 (28.6)	6 (22.2)	
***Clinical characteristic***			
Chronic diseases, n (%)			
Hypertension	20 (71.4)	17 (63.0)	0.573
Diabetes	16 (57.1)	9 (33.3)	0.106
Hyperlipidaemia	11 (39.3)	13 (48.1)	0.591
Medication intake, n (%)			
Anti-diabetics	11 (47.8)	8 (29.6)	0.403
Statins	13 (56.5)	12 (44.4)	0.423
Angiotensin converting Enzyme-inhibitors	8 (34.8)	6 (22.2)	0.375
Calcium channel blockers	10 (43.5)	12 (44.4)	0.787
SBP, mmHg	131.5 ± 14.0	132.6 ± 13.8	0.764
DBP, mmHg	74.9 ± 10.5	76.2 ± 8.8	0.632
PP, mmHg	67.4 ± 11.3	71.7 ± 9.8	0.133
FMD, mm	5.626 ± 1.360	5.368 ± 0.977	0.422
CIMT, mm	0.879 ± 0.232	0.884 ± 0.170	0.920

CIMT, carotid intima-media thickness; DBP, diastolic blood pressure; FMD, flow mediated dilatation; PP, pulse pressure; SBP, systolic blood pressure ^*a*^ Independent-sample Student’s t-tests.

### 3.2 Air pollution exposure

Table 2 shows the indoor and outdoor air pollution exposure levels between the sham- and true-purification groups. Compared with the sham-purification group, the concentrations of indoor PM_2.5_ were significantly reduced in the true-purification group (31.6 μg/m^3^ vs. 22.8 μg/m^3^, P < 0.001), with a particle removing efficiency of 27.8%. The levels of outdoor air pollutants were comparable between the sham- and true-purification groups. In addition, the indoor temperature (23.6 C^o^ [standard deviation: 4.0] vs. 23.7 C^o^ [3.8], p>0.05) and relative humidity (RH) (64.1% [4.9] vs. 63.7% [4.2], p>0.05) between the sham- and true-filtration groups showed no significant differences during the intervention period. Figure S1 shows the variations of the monthly averaged indoor temperature in the randomized groups among the participants completing at least two health measurements.

**Table 2:** Indoor and outdoor air pollutant concentrations of all participants completing at least two health measurements post-randomization.

Variables	Sham-purification		True-purification		P value
	N^a^	Mean ± SD	N^a^	Mean ± SD	
**Indoor**					
PM_2.5_, μg/m^3^	176,842	31.6 ± 42.2	180,827	22.8 ± 35.9	<0.001
**Outdoor**					
PM_2.5_, μg/m^3^	360	18.2 ± 5.6	341	18.2 ± 6.0	0.954
PM_10_, μg/m^3^	360	30.3 ± 9.2	341	29.8 ± 9.5	0.468
NO_2_, μg/m^3^	360	40.0 ± 10.4	341	39.0 ± 10.6	0.221
O_3_, μg/m^3^	360	53.3 ± 18.3	341	53.2 ± 17.4	0.940
SO_2_, μg/m^3^	360	5.7 ± 1.9	341	5.6 ± 2.2	0.684

NO_2_, nitrogen dioxide; O_3_, ozone; PM_2.5_, particulate matter with a mean aerodynamic diameter ≤2.5 μm; PM_10_, particulate matter with a mean aerodynamic diameter ≤10 μm; SO_2_, sulphur dioxide.

a Number of observations. Mean of indoor PM_2.5_ was based on hourly data and mean of outdoor air pollution were based on monthly data.

**Figure 2:**
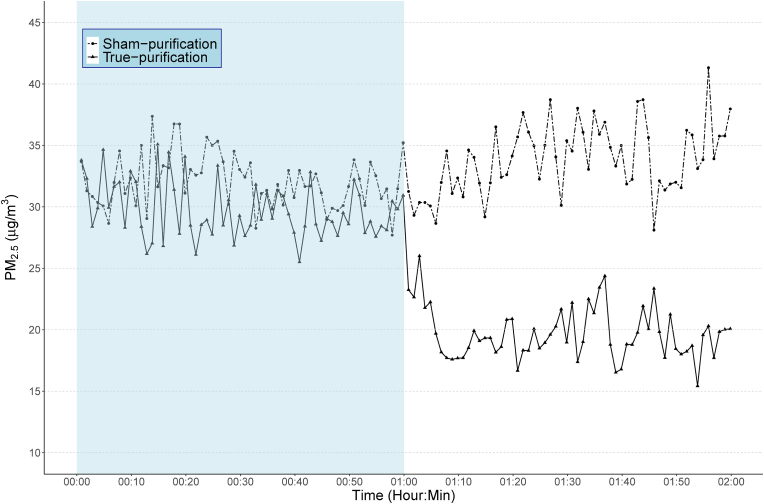
Aggregated time-series plot of indoor PM_2.5_ levels in one hour before and after the operation of air purifiers for participants completing at least two health measurements post-randomization.

Figure 2 shows the aggregated time-resolved indoor PM_2.5_ concentrations during one hour before and after the operation of the IAPs among participants completing at least two health measurements post-randomization. While the indoor PM_2.5_ levels among the sham-purification group remained largely stable during the two hours examined, the levels among the true-purification group dropped promptly and significantly when the IAP was turned on, and remained significantly lower than the sham-purification group thereafter.

**Figure 3:**
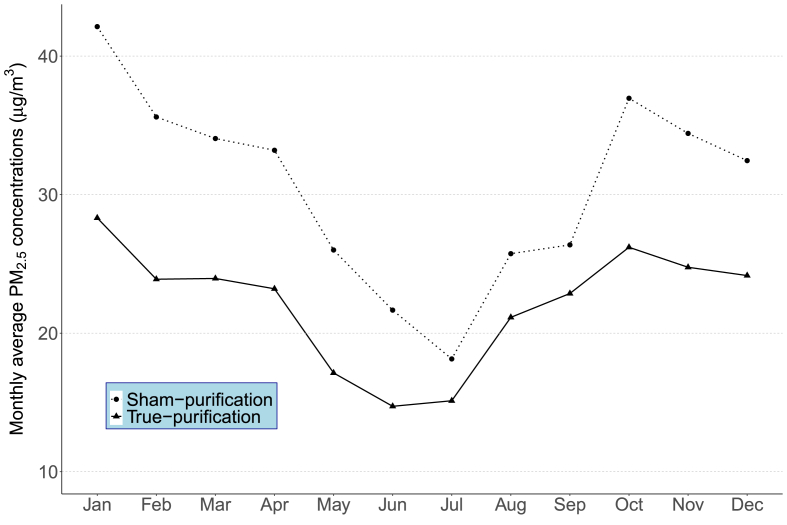
Variation of monthly average PM_2.5_ concentrations in the randomized groups during the intervention periods.

Figure 3 shows the variations of the indoor PM_2.5_ concentrations in the randomized groups during the intervention period, plotted using monthly mean values averaged from all participants completing at least two health measurements. Compared with the sham-purification group, the indoor PM_2.5_ levels were consistently lower in the true-purification group throughout the year, but the differences were smaller during the warm season, when both groups recorded relatively low indoor PM_2.5_ levels.

### 3.3 Health measurements and estimated effects of intervention

The summary for the health measurements statistics across the 1-year intervention period between the sham- and true-air purification groups are shown in Table 3. There was no statistically significant difference in the health outcomes over the 1-year period in the sham-purification group, but the true-purification group showed an indication of reduced DBP (paired t-test, P-value = 0.052).

**Table 3:** Clinical characteristics at baseline, and after 6 months and 12 months intervention of all participants in the randomized groups (arithmetic mean ± SD).

Variables	Sham-purification				True-purification			
	Baseline (n=30)	6-month (n=28)	12-month (n=23)	P value a	Baseline (n=30)	6-month (n=27)	12-month (n=24)	P value a
SBP, mmHg	131.5 ± 14.0	134.1 ± 13.8	140.3 ± 14.0	0.281	132.6 ± 13.8	135.2 ± 20.9	137.6 ± 17.0	0.310
DBP, mmHg	74.9 ± 10.5	77.8 ± 7.7	79.4 ± 5.0	0.737	76.2 ± 8.8	75.3 ± 7.4	75.4 ± 7.9	0.052
PP, mmHg	67.4 ± 11.3	70.1 ± 12.2	72.7 ± 15.8	0.720	71.7 ± 9.8	72.5 ± 10.6	70.8 ± 9.0	0.188
FMD, %	5.653 ± 1.378	5.835 ± 1.339	6.227 ± 1.322	0.466	5.349 ± 0.991	5.520 ± 0.950	5.530 ± 0.679	0.159
CIMT, mm	0.903 ± 0.251	/	0.906 ± 0.252	0.992	0.866 ± 0.155	/	0.866 ± 0.157	0.965

CIMT, carotid intima-media thickness; DBP, diastolic blood pressure; FMD, flow mediated dilatation; PP, pulse pressure; SBP, systolic blood pressure.

a p value refers to within-group changes from baseline to 12 months (paired Students t-test).

After 6 months of intervention, DBP appeared to decrease by 0.9 mmHg in the true-purification group, but increased by 2.9 mmHg in the sham-purification group, although the difference was not statistically significant (P=0.17), and there was no evidence of between-group differences in changes of SBP, PP, and FMD from baseline (Table 4). After 12 months of intervention, the one-way ANCOVA demonstrated a significant reduction of DBP in the true-purification compared with the sham-purification group (-0.3 mmHg vs. 4.6 mmHg, p = 0.033), reflecting a slower, if not largely mitigated, secular trend of increasing BP in relation to the IAP use. Similar patterns were also observed for PP and CIMT, although these were not statistically significant. For SBP, both groups showed increased levels after 12 months, but the average increment appeared lower in the true-purification than the sham-purification group.

**Table 4:** Comparison of absolute changes after 6 months and 12 months of intervention of participants who have completed at least two health measurements post-randomization (arithmetic mean ± SD).

Variables	Δ_6-month_^a^			Δ_12-month_^b^		
	Sham-purification (n=28)	True-purification (n=27)	P value c	Sham-purification (n=23)	True-purification (n=24)	P value d
SBP, mmHg	2.6 ± 19.8	1.4 ± 19.6	0.966	9.4 ± 18.9	5.5 ± 22.8	0.537
DBP, mmHg	2.9 ± 12.3	-0.9 ± 8.7	0.170	4.6 ± 10.1	-0.3 ± 11.2	0.033*
PP, mmHg	2.7 ± 9.8	0.8 ± 9.4	0.841	5.5 ± 10.0	-0.9 ± 9.1	0.054
FMD, %	0.23 ± 0.31	0.15 ± 0.41	0.355	0.45 ± 0.46	0.39 ± 0.76	0.307
CIMT, mm	/	/	/	0.004 ± 0.011	-0.000 ± 0.040	0.745

CIMT, carotid intima-media thickness; DBP, diastolic blood pressure; FMD, flow mediated dilatation; PP, pulse pressure; SBP, systolic blood pressure.

**Figure 4:**
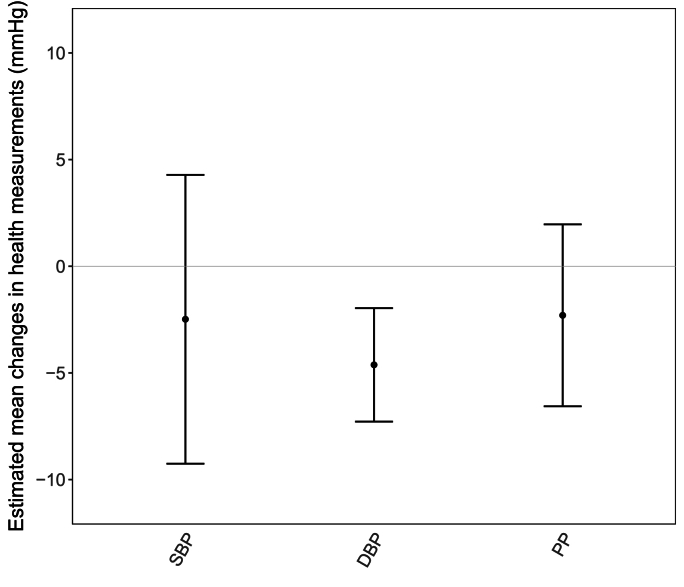
Estimated mean changes (mean, 95% CI) in SBP, DBP and PP with the true-purification intervention.

* p < 0.05.

a Δ6-month = 6 months – baseline.

b Δ12-month = 12 months – baseline.

c p value refers to the difference in changes from baseline to 6 months between the groups (one-way ANCOVA);

d p value refers to the difference in changes from baseline to 12 months between the groups (one-way ANCOVA);

LME models were performed to estimate the effects of air purification for the repeatedly measured health outcomes among the participants who have attended at least two of the three health assessments (i.e., 1^st^, 2^nd^ and 3^rd^ clinical visits), involving 102 data points in each model. As shown in Figure 4, the true-purification was significantly associated with a reduction of 4.62 (95% CI: -7.28, -1.96) mmHg in DBP. Similar magnitude of reduction was also found in SBP (-2.49 [95% CI: -9.25, 4.28] mmHg) and PP (-2.30 [95% CI: -6.57, 1.96] mmHg), although these reductions were not statistically significant. For FMD, true-purification resulted in a non-significant reduction (-0.15 % [95% CI: -0.36, 0.06]). Similarly, the intervention was associated with a non-significant reduction in CIMT (-10.0% [95% CI: -24.8, 4.7]). Table S1 presents the estimated mean changes in SBP, DBP, PP, FMD per IQR higher indoor PM_2.5_ from the LME models. Coherent with the main analyses, PM_2.5_ was found to be positively associated with DBP (1.89 [95% CI: 0.14, 3.64] mmHg per IQR increase of PM_2.5_).

## 4 Discussion

In this trial, we investigated for the first time the long-term cardiovascular effects of IAP intervention among elderly. As expected, the use of indoor HEPA air purifiers can significantly reduce indoor PM_2.5_ levels (by ∼30%) even in a real-world setting, in contrast with many (but not all) previous short-term RCTs with more stringent restrictions on the environments or activities. Such benefits, after sustained 1-year use of IAP in our study sample translated into a significant reduction in DBP and apparent slower secular trends of increasing DBP and SBP. However, we observed no statistically significant changes in other cardiovascular biomarkers (i.e., SBP, PP, FMD, and CIMT).

### 4.1 Blood pressure and pulse pressure

Elevated BP is one of the most important independent risk factors for CVDs (Lewington et al., 2002), and ample evidence supported PM_2.5_ to be a risk factor of increased BP (Allen et al., 2011; Dong et al., 2019; Cui et al., 2018). In this study, a significant decrease in DBP (-4.62 [95% CI: -7.28, -1.96] mmHg), a non-significant reduction in SBP (-2.49 [95% CI: -9.25, 4.28] mmHg) and PP (-2.30 [95% CI: -6.57, 1.96] mmHg) were found to be associated with the long-term IAP intervention among elderly aged ≥70 years. Our finding on DBP is in contrast to the existing literature of short-term RCTs, which showed no significant reduction of DBP in recent systematic reviews and meta-analyses (Liu et al., 2022; Xia et al., 2021). However, our observation is consistent with the only published long-term crossover RCT conducted in healthy middle-aged homemakers in Taipei, which reported -3.20 [-4.50, -1.90] mmHg of DBP associated with true-purification intervention (Chuang et al., 2017). Additionally, our findings show a normal progression of increasing DBP (and SBP) in the sham-purification group as participants aged, whereas such secular trend appeared slowed or even mitigated in the true-purification group. Together, it is indicative that sustained reduction of exposure to indoor PM_2.5_ over a longer-term may be required to reduce DBP.

Despite having a point estimate of reduced SBP comparable to previous (mostly short-term) trials (Xia et al., 2021), the non-significant reduction may reflect a suboptimal noise-to-signal ratio due to multiple reasons. First, SBP is much more variable compared to DBP, as observed in the present trial and previous literature (Mancia et al., 2018a, 2018b). Second, although we have adjusted for self-reported medication usage, we had no details on participants’ hypertension control status. Given the high prevalence of hypertension (∼66%) and the relatively small sample size, unaccounted variations in hypertension management across the intervention and control arms may reduce the precision of the effect estimates. Moreover, due to practical constraints, this long-term RCT was designed as a parallel study instead of crossover RCT, the latter of which could booster the statistical power and reduce noise by allowing the participants to experience both intervention and control phase in a random order, while maintaining a relatively small sample size. Nevertheless, with a point estimate of reduced SBP comparable to previous trials, together with the significant effects on DBP and the apparent slower secular trend of increasing BP, our study lends further support for the long-term benefits of IAP on BP control in the context of PM_2.5_ pollution.

### 4.2 Flow-mediated dilation

The dysfunction of vascular endothelium is the earliest subclinical stage in the pathogenesis of atherosclerotic disease, one of the leading causes of morbidity and mortality globally (Harris et al., 2010; Sans et al., 1997). A recent review indicated endothelial function measurements to potentially better predict future CVD than traditional risk factors(Green et al., 2011). With growing epidemiological evidence linking PM_2.5_ exposure and endothelial dysfunction(Riggs et al., 2019; Zhang et al., 2016), we hypothesised that IAP intervention may alleviate endothelial dysfunction and reduce risk of CVDs. Previous RCTs assessed RHI as an indicator of endothelial function, but we employed a more reliable and conventional method to measure FMD (Onkelinx et al., 2012).

This is the first RCT to examine the effects of long-term IAP intervention on endothelial function (as measured by FMD), but no significant change in FMD was observed. This is somewhat inconsistent with the limited existing evidence from short-term RCTs (Bräuner et al., 2008; Kajbafzadeh et al., 2015; Karottki et al., 2013; Allen et al., 2011; Weichenthal et al., 2013), with our previous meta-analysis showing small but significant improvement in RHI (0.13 [95% CI: 0.01, 0.25]) in RCTs using HEPA-filter air purifiers (Xia et al., 2021). The inconsistency may be mainly due to the small PM_2.5_ exposure difference in this RCT, which observed a more modest particle removing efficiency (∼28%) compared to previous short-term RCTs (40–63%). While our trial was conducted in real-world residential settings with relatively large residential space (>40m^2^) and limited scope of restriction of participants’ behaviour (including closing windows) due to their elderly status, many previous short-term RCTs involved only young healthy adults and were able to imposed more stringent restrictions than the present trial, such as having participants home-bound in small university dormitory (<20m^2^) and closed windows.

### 4.3 Carotid intima-media thickness

Growing evidence from epidemiological studies has demonstrated that long-term exposure to PM_2.5_ is significantly positively associated with CIMT (Adar et al., 2013; Bauer et al., 2010; Kim et al., 2014), an important long-term biomarker for atherosclerosis and subclinical vascular impairment and independent predictor of major CVDs (Oord et al., 2013; Simon et al., 2010, 2002). However, due to the short-term nature of previous RCTs, no previous trials have investigated the potential benefits of IAP on CIMT. In our study, with an average reduction of 8.8 μg/m^3^ in indoor PM_2.5_ levels, there was a non-significantly slower progression rate of CIMT for the participants in the true-purification group when compared with those in the control group (i.e., -0.000 ± 0.040 mm vs. 0.004 ± 0.011 mm), and similar non-significant proportional reduction (-10.0% [95% CI: -24.8%, 4.7%]) was found in the multivariable model. While sample size and the aforementioned limitations of our study may contribute to the imprecise estimate, CIMT is known to progress slowly over a long period of time, and our 1-year intervention in elderly may not be sufficient to capture the benefits of reduced PM_2.5_ exposure. Future trials with a longer intervention period and larger sample size are recommended to investigate CIMT and clarify the treatment effect, as it is a more robust intermediate endpoint for CVD risk compared to other biomarkers (e.g., BP, RHI).

### 4.4 Strengths and limitations

To the best of our knowledge, this is the first study to investigate the long-term cardiovascular benefits of IAP intervention in elderly in real-world residential settings. Despite the relatively small sample size, we reported indicative evidence on the potential benefits of IAP that could be better clarified in future larger-scale long-term RCTs. Furthermore, the inclusion of elderly (mean age >80 years) who tend to be physically less active naturally ensured a high proportion of time spent in filtered air. Our experience also demonstrates the feasibility (with relatively low attrition despite the long intervention period and high-risk elderly subjects) and identified key areas of improvements for future trials.

Our study has some limitations. First, in real-world residential settings with a relatively large average living space, the use of a single IAP in our long-term study inevitably resulted in lower overall residential particle removal efficiency compared to many previous short-term trials with more stringent restrictions in time, space, and/or participants’ behaviours (e.g., small dormitories of <20 m^2^, all windows shut for 48-hours, mandated stay inside the room during the short study period) (Xia et al., 2021). Further, the use of the IAP in the living room alone may also hamper the effect on reducing total personal PM_2.5_ exposure, as participants were likely to spend considerable time in their bedrooms. This may have reduced the effect sizes of the cardiovascular benefits associated with long-term IAP use. Second, the long-term and real-world nature of the study may result in greater confounding by non-adherence or behavioural variability (e.g., opening windows) compared to well-controlled short-term trials. For example, although exposure to major sources of indoor air pollution was reasonably balanced between the true- and sham-purification groups, unmeasured ventilation characteristics may not be balanced. This is particularly likely in the warm season, when the use of air-conditioners, electric fans, and windows differ substantially from the cool season. However, it was reassuring that the indoor PM_2.5_ levels across the two groups were largely consistent, and diverged sharply after the initiation of the IAP. On the same note, we did not collect data on time participants spent at home, but according to previous studies in similar populations, elderly tend to spend 80–90% of their time at home (Chan et al., 2021). Third, as the participants were all elderly (mean age >80), natural attrition due to movement to nursing home or death inevitably reduced our statistical power and prevented us from conducting intention-to-treat analyses. Fourth, the relatively small sample size limited our statistical power and may result in an imbalance of unmeasured confounders and imprecise effect estimates, especially for health outcomes with greater variability. Fifth, we approximated participants’ outdoor PM_2.5_ exposure from fixed-site monitoring stations, and there could be considerable measurement error. Moreover, as all participants lived in high-rise buildings, their actual exposure to outdoor ground-level traffic-related air pollution could be lower than that recorded in the local monitoring network.

### 4.5 Conclusion

In conclusion, as the first study that examined the long-term health effects of IAP on the cardiovascular system among elderly, we found indicative evidence supporting potential cardiovascular benefits of IAP use, most likely via BP reduction, a slower secular trend of increasing BP, or improvements in BP control. More importantly, this trial provides a study framework and experience to inform future trials with larger sample sizes and longer intervention period, which are essential to clarify the long-term cardiovascular effects of IAP. Future trials should also involve health economics analysis to assess the health and environmental costs and benefits of IAP use.

## Funding

Ka Hung Chan acknowledges support from the BHF Centre of Research Excellence, University of Oxford (RE/18/3/34214).

## CRediT authorship contribution statement

**Xi Xia:** Writing – review & editing. **Ka Hung Chan:** Writing – review & editing, Supervision, Methodology, Formal analysis, Data curation. **Timothy Kwok:** Resources, Project administration, Methodology. **ShaoWei Wu:** Writing – review & editing, Methodology. **Chung Ling Man:** Visualization, Project administration, Investigation. **Kin-Fai Ho:** Supervision, Resources, Project administration, Methodology, Investigation, Funding acquisition, Conceptualization.

## Declaration of competing interest

The authors declare the following financial interests/personal relationships which may be considered as potential competing interests:Ka Hung Chan reports financial support was provided by BHF Centre of Research Excellence. If there are other authors, they declare that they have no known competing financial interests or personal relationships that could have appeared to influence the work reported in this paper.

## Data Availability

Data will be made available on request.
