# Supplementary material for: Effects of long-term indoor air purification intervention on cardiovascular health in elderly: a parallel, double-blinded randomized controlled trial in Hong Kong
Source: Environ Res. 2024 Apr 15;247:118284. doi: 10.1016/j.envres.2024.118284 (PMC11850294; doi:10.1016/j.envres.2024.118284)
Supplement: Multimedia component 1 [file mmc1.docx]

**Supplementary file**

**Effects of long-term indoor air purification intervention on cardiovascular health in elderly: a parallel, double-blinded randomized controlled trial in Hong Kong**

Xi Xia ^a b c d^, Ka Hung Chan ^e f*^, Timothy Kwok ^g h^, ShaoWei Wu ^a b^, Chung Ling Man ^d^,

Kin-Fai Ho ^d*^

^a^ Department of Occupational and Environmental Health, School of Public Health, Xi’an

Jiaotong University Health Science Center, Xi’an, Shaanxi, China

^b^ Key Laboratory of Environment and Genes Related to Diseases, Ministry of Education

^c^ School of Public Health, Shaanxi University of Chinese Medicine, China

^d^ The Jockey Club School of Public Health and Primary Care, The Chinese University of Hong

Kong, Hong Kong SAR

^e^ Clinical Trial Service Unit and Epidemiological Studies Unit, Nuffield Department of

Population Health, University of Oxford, UK

^f^ Oxford British Heart Foundation Centre of Research Excellence, University of Oxford, UK

^g^ Department of Medicine and Therapeutics, The Chinese University of Hong Kong, Hong

Kong, China

^h^ The Jockey Club Centre for Osteoporosis Care and Control, The Chinese University of Hong

Kong, Hong Kong, China

*Corresponding authors:

Kin-Fai Ho, *BSc, MPhil, PhD*Professor

Email: [kfho@cuhk.edu.hk](mailto:kfho@cuhk.edu.hk)
JC School of Public Health and Primary Care
The Chinese University of Hong Kong
Hong Kong SAR

Ka Hung Chan, *BSc, MSc, DPhil*
Email: [kahung.chan@ndph.ox.ac.uk](mailto:kahung.chan@ndph.ox.ac.uk)

Oxford BHF CRE Intermediate Transition Fellow
Nuffield Department of Population Health
University of Oxford
United Kingdom

| **Table S1**. Estimated mean changes in SBP, DBP, PP, FMD per interquartile range change higher indoor PM_2.5_ from the linear mixed-effect models | | | | |
| --- | --- | --- | --- | --- |
|  | **Mean change** | | **95% CI** | |
| SBP, mmHg | | 1.81 | | -2.30, 5.92 |
| DBP, mmHg | | 1.89 | | 0.14, 3.64 |
| PP, mmHg | | 1.54 | | -1.73, 4.81 |
| FMD, % | | 0.16 | | -0.14, 0.47 |
| DBP, diastolic blood pressure; FMD, flow mediated dilatation; PP, pulse pressure; SBP, systolic blood pressure. | | | | |

**Figure S1.** Variation of monthly average indoor temperature in the randomized groups during the intervention periods.
